# Increase of Ca_V_3 channel activity induced by HVA β1b-subunit is not mediated by a physical interaction

**DOI:** 10.1186/s13104-018-3917-1

**Published:** 2018-11-14

**Authors:** Rogelio Arteaga-Tlecuitl, Ana Laura Sanchez-Sandoval, Belen Ernestina Ramirez-Cordero, Margarita Jacaranda Rosendo-Pineda, Luis Vaca, Juan Carlos Gomora

**Affiliations:** 1 Departamento de Neuropatología Molecular, Instituto de Fisiología Celular, Universidad Nacional Autónoma de México, 04510 Mexico City, Mexico; 2 Departamento de Biología Celular y del Desarrollo, Instituto de Fisiología Celular, Universidad Nacional Autónoma de México, 04510 Mexico City, Mexico

**Keywords:** Voltage-gated calcium channel, Ca_V_3 channel, Electrophysiology, β-Subunit, Protein–protein interaction, Fluorescence, Colocalization, FRET

## Abstract

**Objective:**

Low voltage-activated (LVA) calcium channels are crucial for regulating oscillatory behavior in several types of neurons and other excitable cells. LVA channels dysfunction has been implicated in epilepsy, neuropathic pain, cancer, among other diseases. Unlike for High Voltage-Activated (HVA) channels, voltage-dependence and kinetics of currents carried by recombinant LVA, i.e., Ca_V_3 channels, are quite similar to those observed in native currents. Therefore, whether these channels are regulated by HVA auxiliary subunits, remain controversial. Here, we used the α1-subunits of Ca_V_3.1, Ca_V_3.2, and Ca_V_3.3 channels, together with HVA auxiliary β-subunits to perform electrophysiological, confocal microscopy and immunoprecipitation experiments, in order to further explore this possibility.

**Results:**

Functional expression of Ca_V_3 channels is up-regulated by all four β-subunits, although most consistent effects were observed with the β1b-subunit. The biophysical properties of Ca_V_3 channels were not modified by any β-subunit. Furthermore, although β1b-subunits increased colocalization of GFP-tagged Ca_V_3 channels and the plasma membrane of HEK-293 cells, western blots analysis revealed the absence of physical interaction between Ca_V_3.3 and β1b-subunits as no co-immunoprecipitation was observed. These results provide solid evidence that the up-regulation of LVA channels in the presence of HVA-β1b subunit is not mediated by a high affinity interaction between both proteins.

**Electronic supplementary material:**

The online version of this article (10.1186/s13104-018-3917-1) contains supplementary material, which is available to authorized users.

## Introduction

Voltage-gated calcium (Ca_V_) channels play a crucial role in cell Ca^2+^ influx, which in turn influences several cell functions as cellular excitability, muscle contraction, hormone and neurotransmitter secretion, and gene expression [1]. The Ca_V_ channels family is classified in low- and high-voltage activated (LVA and HVA) channels based on their activation threshold [2]. The conduction pore of these channels is formed by the α1-subunit, a four homologous domains (I–IV) single protein [3]. Auxiliary subunits, named β, α2δ and γ, modulate the activity of HVA channels [4, 5]. In particular, β-subunits, modulate HVA channels by increasing their surface expression [6–9], and modifying the voltage-dependence and current kinetics [reviewed by Refs. 4, 6]. α1- and β-subunits interaction takes place through the AID (alpha interaction domain) motif, localized at the intracellular link between domain I and II of α-1-subunits, and the alpha-binding pocket (ABP) site of β-subunits; this is a high-affinity interaction ranging from 2 to 54 nM [10–12]. In addition, low-affinity interaction sites at the amino and carboxy termini of HVA channels have also been implicated [13–16]. In contrast, it has been suggested that LVA channels (also known as T-type or Ca_V_3 channels), are not modulated by HVA auxiliary subunits [17–19]. LVA channels are responsible for the low-threshold Ca^2+^ spikes in several central nervous system neurons [20], and they have also been implicated in pathophysiological conditions such as epilepsy, neuropathic pain, neuropsychiatric disorders and cancer [21–24]. Native LVA Ca^2+^ currents show an electrophysiological behavior quite similar to that of recombinant channels expressed without auxiliary subunits [25, 26], and their subunit composition is unknown due to the in silico strategy they were cloned with [25, 27–29]. Nevertheless, some studies support the notion that auxiliary subunits [30–33] might regulate Ca_V_3 channels. More recently, a low-affinity association between synthetic peptides of Ca_V_3.3 I-II loop and HVA β-subunits has been suggested [34]. Here, we addressed whether full-length Ca_V_3 channel proteins and HVA β-subunits interact physically; our results provide experimental evidence that β-subunits up-regulate current density and the number of Ca_V_3 channels in the plasma membrane by a mechanism that does not involve strong physical interactions between them, but rather they might have a low-affinity interaction.

## Main text

### Methods

#### Cell culture and transfection

HEK-293 cells were grown in DMEM/F12 mixture supplemented with 10% FBS, 100 U/ml penicillin, and 100 μg/ml streptomycin at 37 °C with 5% CO_2_. Transient transfections were performed with JetPei (polyplus-transfection) in 35-mm dishes, according to manufacturer’s protocol. Transfections were done with 1.5 µg of main α-subunit coding for Ca_V_1.2 (GenBank accession #AY728090), Ca_V_3.1 (#AF190860), Ca_V_3.2 (#AF051946), Ca_V_3.3 (#AF393329), or Na_V_1.6 (#NM_019266); 1.5 µg of the auxiliary β-subunit coding for Ca_V_β1a (M25817), Ca_V_β1b (X61394), Ca_V_β2a (M80545), Ca_V_β3 (X64300) and Ca_V_β4 (L02315); and 0.2 µg of GFP. Cells were dissociated 24-72 h after transfection and plated on coverslips for electrophysiological experiments.

#### Electrophysiology

Whole-cell Ca^2+^ (and Na^+^) currents were recorded at room temperature (21–23 °C) with the patch-clamp technique following the methods of Sanchez-Sandoval et al. [35]. External solutions composition was as follows (in mM): for LVA channels, 5 CaCl_2_ and 175 TEA-Cl; for HVA channels, 10 BaCl_2_ and 152 TEA-Cl; and for sodium channels, 158 NaCl, 2 CaCl_2_, and 2 MgCl_2_. Borosilicate glass pipettes (WPI Inc.) with resistances of 2–3 MΩ were filled with an internal solution containing (in mM): 130 CsCl, 10 EGTA, 2 CaCl_2_, 1 MgCl_2_, 4 Mg-ATP, and 0.3 Tris-GTP, for Ca_V_ channels; or with 106 CsCl, 30 NaCl, 1 CaCl_2_, 1 MgCl_2_ and 10 EGTA, for Na_V_ channels. All solutions contained also 10 HEPES and were adjusted to pH 7.3 with TEA-OH, NaOH or CsOH, accordingly. Current recordings were analyzed using Clampfit software (Molecular Devices). Quantitative results are given as the mean ± standard error (SEM).

#### Construction of α1 and β-subunits of Ca_V_ channels tagged with fluorescent proteins

Fusion proteins were constructed by introducing restriction enzyme sites through the PCR mutagenesis technique. To generate Ca_V_3.1-GFP and Ca_V_3.2-GFP channels, the respective α1-subunits were cloned at the 3′ end of GFP in the multiple cloning site of pEGFP-C1 vector. The Ca_V_3.3 channel with the GFP fused in the N-terminal and tagged with the hemagglutinin (HA) epitope in the extracellular S1–S2 of domain I [36], was modified to delete the HA epitope but conserving the GFP (Ca_V_3.3-GFP). The Ca_V_1.2 channel and the β1b-subunit were N-terminal tagged with the GFP and the BFP, by using the nucleotide sequence of the pRSET/GFP and pRSET/BFP vectors, respectively (Invitrogen). All constructions were verified by automated sequencing.

#### Confocal microscopy

For subcellular localization of GFP-tagged α1-subunits, HEK-293 cells were transfected with the corresponding α1-subunits alone or with the BFP-β1b subunit in 12 well-plates using 1.25 µg of each DNA subunit and PEI (Santa Cruz Biotechnology) as transfection reagent. Forty-eight hours after transfection the cells were cultured in 25-mm diameter coverslips for 12 h. Plasma membrane of HEK-293 cells was stained with FM4-64 (Invitrogen). Images were collected with an Olympus Fv10i confocal microscope equipped with a UPLSAPO 60×/1.35 oil immersion objective and using the following filters: for GFP-tagged channels the exciting wavelength was 489 nm; for β1b-BFP was 405 nm, and for FM4-64 was 559 nm. The confocal acquisition window was set to 512 × 512 pixels which allowed to acquire one image every 9 s for each fluorophore. Pearson colocalization coefficients where calculated with Imaris 8.2 software (Bitplane).

#### FRET measurements by sensitized emission

The Förster resonance energy transfer (FRET) measurements between the α1-subunits from Ca_V_ channels and the β1b subunit were obtained using the sensitized emission (SE) protocol. Briefly, FRET was obtained by measuring the acceptor emission resulting from donor excitation. To avoid overestimation of FRET first we evaluated the bleed-trough between both fluorescence channels for donor and acceptor. After subtracting the bleed-through from the donor emission we calculate FRET by using the following equation:

$$ nF = F^{{ex_{D} , em_{A} }} - \alpha F^{{ex_{A} ,em_{A} }} - \beta F^{{ex_{D} , em_{D} }} $$

Using an excitation wavelength that excites only the donor, the emission for the acceptor ($F^{{ex_{D} , em_{A} }}$) and donor ($F^{{ex_{D} , em_{D} }}$^**)**^ channels are obtained. Next, fluorescence is measured in the acceptor channel ($F^{{ex_{A} , em_{A} }}$) at an excitation wavelength that only excites the acceptor. The amount of donor bleed-through into the acceptor channel is determined by a donor-only measurement, which provides the calibration constant $\beta = {{F_{D}^{{ex_{D} , em_{A} }}  } \mathord{\left/ {\vphantom {{F_{D}^{{ex_{D} , em_{A} }}  } {F_{D}^{{ex_{D} , em_{D} }} }}} \right. \kern-0pt} {F_{D}^{{ex_{D} , em_{D} }} }}$ By measuring only the acceptor channel we obtained the constant $\alpha = {{F_{A}^{{ex_{D} , em_{A} }} } \mathord{\left/ {\vphantom {{F_{A}^{{ex_{D} , em_{A} }} } {F_{A}^{{ex_{A} , em_{A} }} }}} \right. \kern-0pt} {F_{A}^{{ex_{A} , em_{A} }} }}$, for complete calibration procedures refer to [37]. FRET analysis was performed by using ImageJ with the Fret Analyzer plugin.

#### Co-immunoprecipitation and western blot

Total protein from transfected HEK-293 cells was extracted 48 h post-transfection using RIPA buffer supplemented with complete protease inhibitor (Roche). Co-immunoprecipitation (Co-IP) was performed by mixing total protein extracts from β1b-HA transfected cells with those of Ca_V_1.2-GFP or Ca_V_3.3-GFP, and incubated overnight at 4 °C in the presence of Anti-HA Affinity Matrix (Roche). Briefly, 50 μl of this matrix were washed and mixed with 1 mg of total protein from Ca_V_1.2-GFP or Ca_V_3.3-GFP transfected cells, and 1 mg of total protein from β1b-HA transfected cells. Then, beads were rinsed three times with RIPA buffer and proteins were eluted with Laemmli sample buffer, boiled at 95 °C for 3 min and analyzed by western blotting. The specific antibodies were used as follows: rat anti-HA (1:5000; Roche) for the β1b-HA subunit; and rabbit anti-GFP (1:5000; Santa Cruz Biotechnology) to identify Ca_V_1.2-GFP and Ca_V_3.3-GFP channels. Secondary antibodies were both raised in goat against rat and rabbit IgG-HRP (1:10,000; Santa Cruz Biotechnology). For control experiments, 15 µg of total protein from cell lysates of transfected cells were used in the immunoblots, as well as 10 µl (from a total of 500 µl) of the Co-IP supernatants. For loading control, a homemade monoclonal antibody against human β-actin was used (donated by Dr. Manuel Hernandez, CINVESTAV, Mexico).

### Results

#### Ca_V_3 channels current density increases in the presence of β1b

**Fig. 1:**
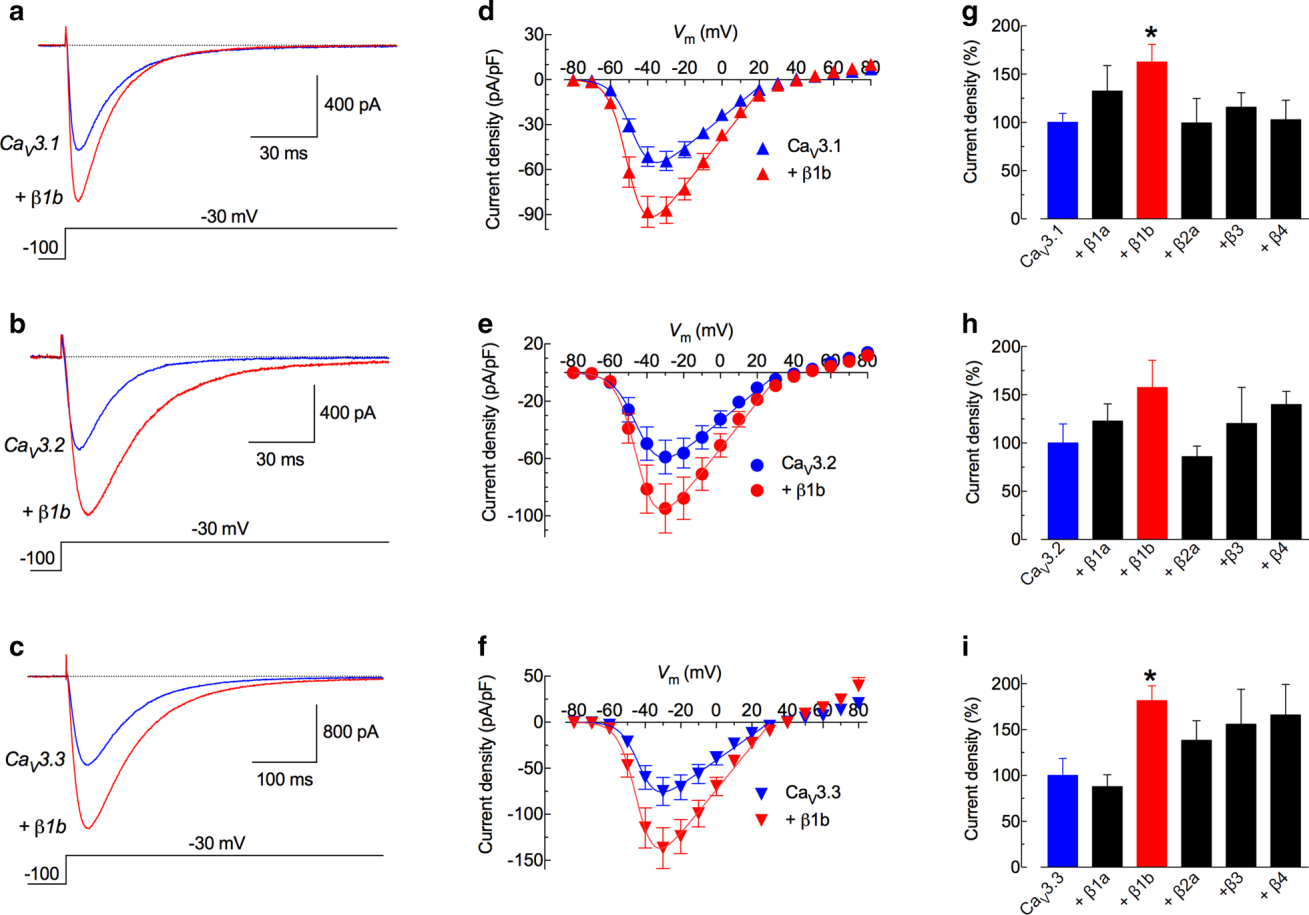
The β1b-subunit increases the current density in all three LVA channels. **a**–**c** Representative whole-cell currents recorded at -30 mV from HEK-293 cells transfected with each of the Ca_V_3 channels (Ca_V_3.1, Ca_V_3.2 or Ca_V_3.3) alone or together with the β1b-subunit. Patch-clamp experiments were performed by using a HP of − 100 mV and 5 mM CaCl_2_ as charge carrier. **d**–**f** Current–voltage (*I*–*V*) relationships for the same channels as in **a**–**c**. For *I*–*V* plots data were obtained from currents evocated from − 80 mV to + 80 mV in 10 mV steps; current amplitudes were normalized by cell capacitance to obtain current density values. Smooth lines are fits to data with a modified Boltzmann function (see Experimental Procedures). The corresponding parameters are shown in Additional file 1. **g**–**i** Current density (mean ± SEM) at − 30 mV calculated for HEK-293 cells transfected with the indicated Ca_V_3 channels alone or together with β1a, β1b, β2a, β3 or β4 subunits. Only β1b increases current density significantly when transfected with any of the Ca_V_3 channels. Data were normalized to the current density values obtained when Ca_V_3 channels were transfected alone. *Statistical significance when using ANOVA followed by Dunnett’s multiple comparison test (*P *< 0.05). The number of studied cells varied from 5 to 29

Ca_V_3.1, Ca_V_3.2 and Ca_V_3.3 channels were co-expressed with each of the five β-subunits (β1a, β1b, β2a, β3 or β4) in HEK-293 cells, and whole-cell Ca^2+^ currents were analyzed by patch-clamp recordings. Figure 1a–c shows representative Ca^2+^ currents recorded at − 30 mV from each of the Ca_V_3 channels in the absence (blue traces) and presence of the β1b-subunit (red traces). In all cases, current amplitudes were larger when β1b was co-transfected with the α1-subunits. The rise in current amplitudes was observed in the whole range of potentials that induced inward currents, without significant changes in the voltage-dependence of activation (Fig. 1d–f). Although all β-subunits increased current density of at least one of the Ca_V_3 channels, only β1b-subunit was able to induce significant increments in Ca_V_3.1 and Ca_V_3.3 channels; however, the amplitude of Ca_V_3.2 currents were not statistically different from the control (Fig. 1g–i). On average, β1b-subunit promoted increments of 63 ± 18, 57 ± 28, and 81 ± 16% in current density of HEK-293-cells transfected with Ca_V_3.1, Ca_V_3.2 and Ca_V_3.3, respectively. Except for a discrete, but significant shift (4 mV) to more negative potentials in the *V*_50_ of voltage-dependence of activation of Ca_V_3.3 currents, there were no additional changes in activation or inactivation channel gating, neither in current kinetics of activation, inactivation or recovery of inactivation due to the presence of the β1b-subunit (see Additional file 1). Like a positive control, we co-transfected the Ca_V_1.2 channel and the β1b-subunit; as expected, the β1b-subunit induced a drastic (4-fold) increase in the current density; whereas no significant effect was observed when co-transfected with a voltage-gated sodium channel (negative control, see Additional file 2). Thus, the co-transfection of β1b-subunit with Ca_V_3 channels induces specific and significant increases in current density without affecting the biophysical properties. Previous studies have shown similar effects [30, 31], although our electrophysiological data for Ca_V_3.3 are the first to clearly show the effect of β-subunits.

#### Ca_V_3 channels cell surface localization is increased when co-expressed with β1b-subunit

**Fig. 2:**
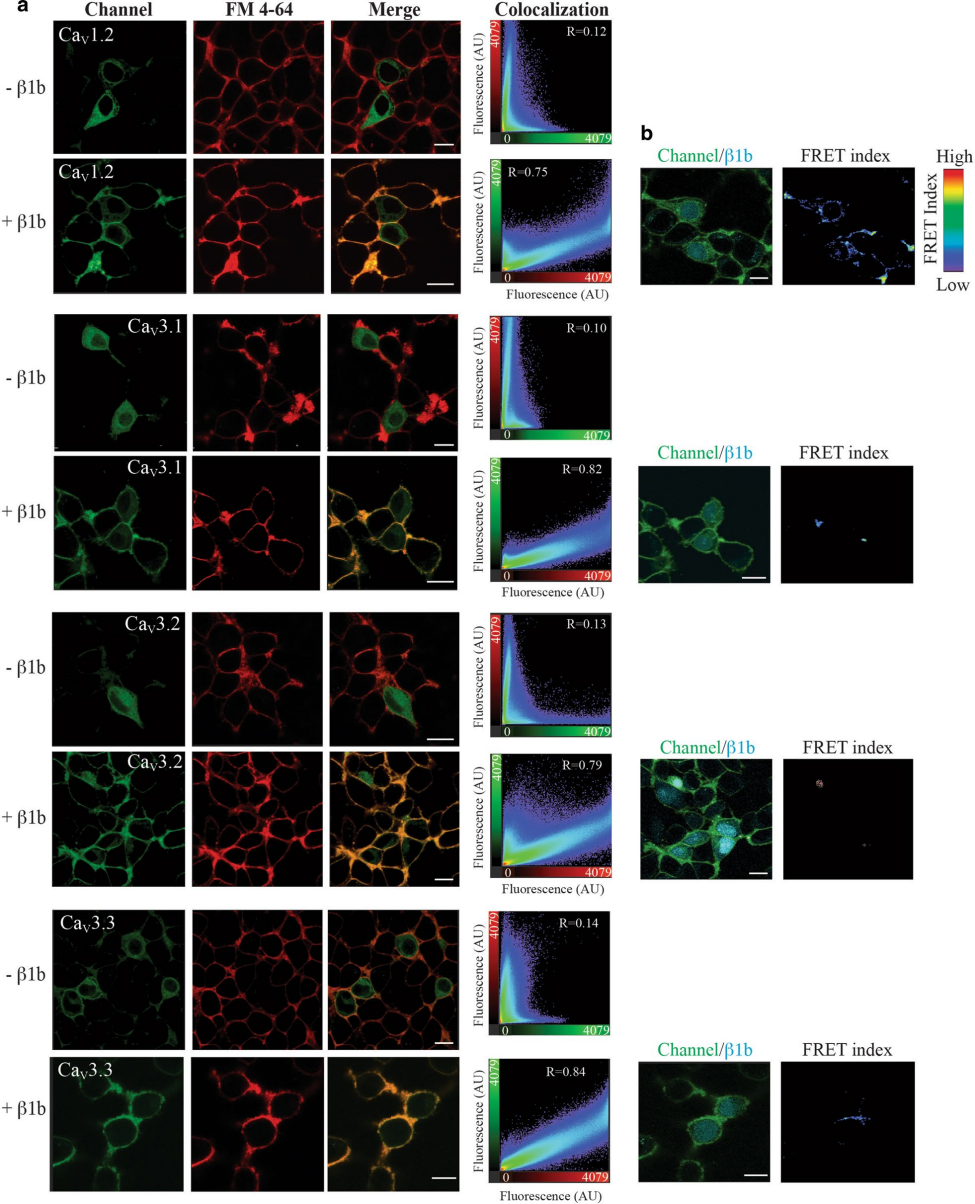
The β1b-subunit increases the amount of Ca_V_ channels at the plasma membrane. **a** Co-localization analysis of HEK-293 cells expressing the α1-subunit of Ca_V_1.2, Ca_V_3.1, Ca_V_3.2 and Ca_V_3.3 fused to GFP, in the absence (-β1b) and presence (+β1b) of the β1b-subunit. Representative confocal microscopy images of cells expressing the respective Ca_V_ shown in green (left panels) and the plasma membrane marker FM4-64 (shown in red, middle panel). The co-localization between the Ca_V_ α1-subunit and the FM4-64 (in yellow) is shown in Merge panels. The plots to the right show the total pixels of colocalization between the green channel (Ca_V_) and the red channel (FM-464), with the corresponding Pearson’s correlation coefficient (R) for each experimental condition. **b** The FRET between the GFP in each Ca_V_ channel and the blue fluorescent protein in the β1b-subunit. Notice that FRET is observed only between Ca_V_1.2 (HVA channel) and β1b, but not with the other 3 Ca_V_ channels (Ca_V_3.1, Ca_V_3.2 and Ca_V_3.3). High and low FRET was calculated pixel-by-pixel and the image shows in pseudo color FRET intensities. The number of cells analyzed for both colocalization and FRET panels were as follows: Ca_V_1.2, 65; Ca_V_3.1, 35; Ca_V_3.2, 48; and Ca_V_3.3, 32. The cells were obtained from 4 to 13 independent experiments. Scale bar: 10 μm

To determine whether the observed increments in current density of Ca_V_3 channels co-expressed with β1b-subunits were the result of increases channel presence at the plasma membrane of HEK-293 cells, we investigated first the plasma membrane localization of all Ca_V_3 channels by confocal microscopy in the presence or absence of β1b-subunit. For this purpose, we used GFP-tagged Ca_V_1.2 and Ca_V_3 channels, and BFP-tagged β1b-subunit (β1b-BFP). As a reference, plasma membrane was stained with the fluorescent marker FM4-64. Transient transfection of HEK-293 cells with Ca_V_1.2 or Ca_V_3 channels showed a fluorescence signal mainly restricted to the plasma membrane and some intracellular membranes (Fig. 2a; -β1b rows). Interestingly, when α1-subunits of Ca_V_1.2 and Ca_V_3 channels were co-transfected with β1b-BFP the colocalization of all Ca_V_ channels with the plasma membrane marker increased drastically (Fig. 2a, merge and colocalization columns). On average, the Pearson’s coefficient of colocalization between Ca_V_ channels and plasma membrane increased more than sixfold in the presence of β1b-subunit. These results suggest that β1b promotes the trafficking of Ca_V_1.2 (HVA) and Ca_V_3 (LVA) α1-subunits to the plasma membrane, which is consistent with our electrophysiological results shown in Fig. 1. Then, to determine if the increased trafficking of Cav3 to the plasma membrane was the result of a direct physical interaction with the β1b-subunit, we conducted FRET measurements between the α1-subunits from all Ca_V_ channels and the β1b-subunit by using the sensitized emission (SE) protocol [37]. As previously reported [38, 39], β1b showed a wide distribution throughout the cytoplasm and the nucleus (Fig. 2b, channel/β1b panels). When Ca_V_ channels were co-expressed with the β1b-subunit, a clear FRET signal was observed mainly in the plasma membrane of co-transfected cells with Ca_V_1.2 channel (Fig. 2b, Ca_V_1.2 FRET index panels). Most interestingly, no significant FRET was observed for Cav3 channels. Among LVA channels only the Ca_V_3.3 showed a weak FRET signal (Fig. 2b, Ca_V_3.3 FRET index panels). Thus, FRET analysis suggests that Ca_V_1.2 α1-subunit and β1b-subunit are within the range distance of 10–100 nm, but only Ca_V_3.3 channels and β1b- seem to be within the same distance.

#### The Ca_V_3.3 channel does not interact physically with the β1b-subunit

**Fig. 3:**
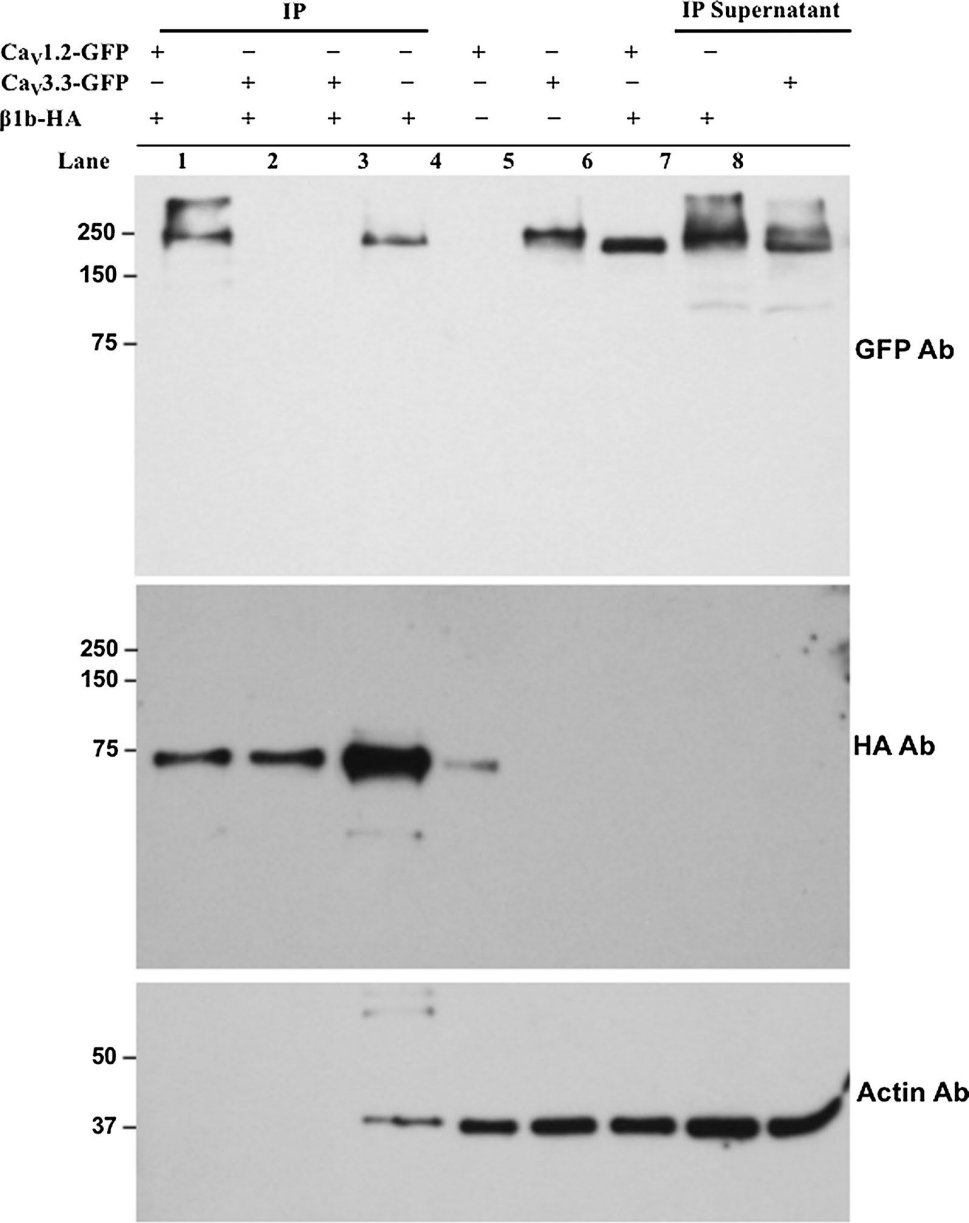
Ca_V_3.3 channels and β1b-subunit do not coimmunoprecipitate. Western blot of IP of Ca_V_1.2-GFP with β1b-HA (positive control, lane 1) and Ca_V_3.3-GFP with β1b-HA (lanes 2 and 3). IPs in lanes 1 and 2 were washed three times with lysis buffer after overnight incubation with the indicated proteins, whereas the IP from lane 3 was washed only once. The immunoblot was probed with an antibody against GFP (upper panel), then striped and probed with an anti-HA antibody (middle panel), then striped again and probed with a homemade anti-actin antibody (lower panel). Lanes 4-6 were loaded with 15 µg of total protein from lysates of HEK-293 cells transfected with β1b-HA, Ca_V_1.2-GFP and Ca_V_3.3-GFP, respectively. Finally, lanes 7 and 8 were loaded with 10 µl of the IP supernatant from lanes 1 and 2, accordingly. Notice the importance of exhaustive washing procedures, since incomplete washing of the beads could lead to false positives results. As can be observed, Ca_V_1.2 channels coimmunoprecipitate with β1b-HA (lane 1, upper panel), whereas Ca_V_3.3 channels do not (lane 2, upper panel). Representative figures of three independent experiments

The potential physical interaction between Ca_V_3.3 channels and β1b-subunits was further investigated with co-immunoprecipitation (Co-IPs) and western blot assays. An expected band of about 75-kDa was clearly detected in total protein extracts from HEK-293 cells transfected with β1b-HA, and from immunoprecipitations of these extracts with an anti-HA affinity matrix (Fig. 3, middle panel), but it was totally absent in extracts of transfected cells with either Ca_V_1.2 or Ca_V_3 channels (Fig. 3, lanes 5 and 6, middle panel), demonstrating the specificity of the HA antibody. Additionally, a ~ 250 kDa band was revealed with the anti-GFP antibody in extracts of HEK-293 cells transfected with the Ca_V_1.2-GFP or Ca_V_3.3-GFP channels (Fig. 3, lanes 5 and 6, upper panel). Furthermore, the Ca_V_1.2-GFP/β1b-HA protein complex was co-immunoprecipitated with the anti-HA affinity matrix and the Ca_V_1.2-GFP was detected as a 250-kDa band using anti-GFP (Fig. 3, lane 1, upper panel). On the contrary, the same procedure did not show any co-immunoprecipitation when Ca_V_3.3-GFP channels were used instead of the HVA channel (Fig. 3, lane 2, upper panel). The supernatants obtained from Co-IPs samples loaded in lanes 1 and 2 showed considerable amounts of Ca_V_1.2 (lane 7) and Ca_V_3.3 (lane 8) channels, indicating that the lack of GFP-immunoreactivity signal in lane 2 was due to the absence of a strong physical interaction between the Ca_V_3.3 channels and the β1b-subunit, rather than a shortage of the protein in the sample. Interestingly, when the Co-IPs were processed less exhaustively, by washing the beads only once instead of three times as done for those in lanes 1 and 2 of Fig. 3 (upper panel), the immunodetection with the GFP antibody revealed a band corresponding to the Ca_V_3.3 channels, and an at least 3-times stronger signal for the β1b-HA-subunit in the same IPs (Fig. 3, lane 3), as well as the presence of actin (Fig. 3, lane 3, lower panel), indicating the importance of correct washing procedures. Altogether, these results suggest that the β1b-subunit does not interact with Ca_V_3.3 channels as strongly (high-affinity) as with HVA Ca_V_1.2 channels, on the contrary, such interaction, if any, is rather weak (low-affinity).

### Discussion

LVA calcium channels display unique functional properties that support critical cell functions in a variety of tissues, and their dysfunction is associated with pathological consequences, such as epilepsy and spinocerebellar ataxia [40–42]. In addition, LVA channel activity is regulated by different cellular mechanisms involving the action of neurotransmitters and hormones [43–45]; and during cellular process like differentiation and proliferation [21, 46]; however, regulation by HVA channels accessory subunits is still a field of controversy. Several reports have shown that LVA calcium channels are not regulated by these auxiliary subunits [17–19, 47]. One of these reports showed that depletion of β-subunits in nodosus ganglion neurons had no significant changes in LVA calcium currents [47]. Nevertheless, these cells do not express the β1b-subunit, which according to our data, induces the most important changes in LVA channels activity. Thus, the results reported by [47] could be due to the absence of β1b-subunit expression in such neurons. In contrast, recent evidence suggest that LVA calcium channels are modulated by HVA accessory subunits [30, 31, 34]. By using in vitro immunoassays, Bae and coworkers [34] suggested a low-affinity interaction between β-subunits and synthetic peptides of Ca_V_3.3 channels containing the equivalent AID sequence (30-residues peptides). Here, we show that co-transfection of Ca_V_3 channels with different β-subunits lead to an increase in current density, an effect that was more consistent and robust with Ca_V_3.3 channels, and to a lesser extent in Ca_V_3.1 and Ca_V_3.2. These effects were limited to current density as biophysical properties of channels were not affected, as previously reported by others [19, 31].

By using full-length α1-subunits of LVA and Ca_V_1.2 channels, we observed a robust increase in colocalization of these α1-subunits with the plasma membrane in the presence of the β1b-subunit. In addition, FRET studies between Ca_V_1.2 channels and β1b-subunit confirm a physical interaction between both proteins. However, FRET was practically absent for Ca_V_3 channels and β1b-subunit. These observations were further confirmed by co-immunoprecipitation experiments where only Ca_V_1.2 channel protein co-immunoprecipitated with the β1b-subunit, suggesting that LVA Ca_V_3.3 channel is not close enough to the β1b-subunit to have a strong physical interaction as the one display by the HVA Ca_V_1.2 channel (Fig. 3).

Thus, the electrophysiological regulation of Ca_V_3 channels by β-subunits shown in Fig. 1 could not be explained by a strong physical interaction between these proteins, but by the increment of cell surface localization of these channels. The lack of a strong interaction between Ca_V_3.3 and the β1b-subunit could be explained by the absence of the AID motif, which has been widely proven to mediate the physical interaction between HVA channels and β-subunits [48–50]. However, non-AID motif interactions between the Ca_V_3 α1-subunits and β-subunits cannot be ruled out. In fact, this possibility is supported by the observation that synthetic Ca_V_2.1-AID peptide did not alter the binding of the Ca_V_3.3-AID peptide to β or β4, suggesting that the β-subunit ABP do not play a role in binding to the Ca_V_3.3-AID [34]. Additional evidence leading to the possibility of multiple interaction sites between HVA α1-subunits and β-subunits include structural studies as well [51, 52]. Because we used the whole proteins for our co-immunoprecipitation experiments, identifying the precise amino acids involved in the interaction is not possible.

In summary, we have found that LVA calcium channels are regulated by the β1b-subunit by increasing membrane channel protein and current density in HEK-293 cells. Nevertheless, low or null FRET signal suggests a weak or null physical interaction between both proteins, which in turn could explain the increment in Ca_V_3 channel membrane density. It is noteworthy that the weakness of this interaction might be the main reason for the discrete, and sometimes, totally absent regulation of the LVA channels expression and biophysical properties.

## Limitations

Our results show the lack of physical interaction between full-length Ca_V_3.3 channels and β1b-subunits, it remains to be explored this issue for the other HVA β-subunits.

## Additional files

**Additional file 1.** Effects of β1b subunit in the biophysical properties of Ca_V_3 channels. Table containing the biophysical properties of Ca_V_3 channels in the absence and the presence of β1b subunit.

**Additional file 2.** Modulation by the β1b subunit is specific on Ca_V_ channels. Electrophysiological recordings and *I*-*V* relationship for HVA Ca_V_1.2 and Na_V_1.6 channels in the absence and the presence of the β1b subunit.

## Abbreviations

HEK-293 cells: human embryonic kidney cells
DMEM: Dulbecco’s Modified Eagle’s Medium
FBS: fetal bovine serum
GFP: green fluorescent protein
TEA: tetra-ethyl-ammonium
PCR: polymerase chain reaction
BFP: blue fluorescent protein
FRET: Förster resonance energy transfer
Co-IP: co-immunoprecipitation
