# Supplementary material for: Increase of CaV3 channel activity induced by HVA β1b-subunit is not mediated by a physical interaction
Source: BMC Res Notes. 2018 Nov 14;11:810. doi: 10.1186/s13104-018-3917-1 (PMC6236959; doi:10.1186/s13104-018-3917-1)
Supplement: Supplementary file 1 — Additional file 1. Effects of β1b subunit in the biophysical properties of CaV3 channels. Table containing the biophysical properties of CaV3 channels in the absence and the presence of β1b subunit. [file 13104_2018_3917_MOESM1_ESM.pdf]

Additional file 1.

Increase of Ca_V3 channel activity induced by HVA β1b-subunit is not mediated by a physical interaction

Rogelio Arteaga-Tlecuitl[1], Ana Laura Sanchez-Sandoval[1], Belen Ernestina Ramirez-Cordero[1], Margarita Jacaranda Rosendo-Pineda[2], Luis Vaca[2], Juan Carlos Gomora[1,*]

[1]Departamento de Neuropatología Molecular and [2]Departamento de Biología Celular y del Desarrollo, Instituto de Fisiología Celular, Universidad Nacional Autónoma de México. Ciudad de México, 04510, México.

[*]Corresponding author: jgomora@ifc.unam.mx

Additional file 1. Effects of β1b subunit in the biophysical properties of Ca_V3 channels.

Channel	I_{Ca} (pA/pF)	Activation V_{50} (mV)	Inactivation V_{50} (mV)	τ_{act} (ms)	τ_{inact} (ms)	τ_h (ms)
$Ca_V3.1$	-55 ± 5	-47.7 ± 0.7	-77.0 ± 0.9	2.5 ± 0.09	16.1 ± 0.7	106 ± 4
$Ca_V3.1$ + β1b	-87 ± 9*	-50.8 ± 0.4	-77.4 ± 0.5	2.1 ± 0.07	16.2 ± 0.5	108 ± 4
$Ca_V3.2$	-55 ± 8	-43.9 ± 1.3	-73.8 ± 1.0	4.8 ± 0.3	22.9 ± 1.4	489 ± 36
$Ca_V3.2$ + β1b	-95 ± 17	-44.4 ± 1.0	-73.0 ± 0.7	5.4 ± 0.4	24.5 ± 2.3	495 ± 19
$Ca_V3.3$	-81 ± 9	-41.9 ± 1.6	-71.5 ± 0.7	17.9 ± 1.2	69.9 ± 3.5	351 ± 14
$Ca_V3.3$ + β1b	-129 ± 11*	-45.9 ± 0.7*	-73.1 ± 0.8	14.6 ± 1.0*	65.9 ± 3.2	345 ± 9

Values are given as mean ± SEM. Current density (pA/pF) at -30 mV. V_{50} values were obtained from *I-V* and SSI data fits with Boltzmann functions. τ_{act} and τ_{inact} were obtained from two exponential fits of current recordings at -30 mV. τ_h was obtained from single exponential fits to the recovery from inactivation data at -100 mV. The number of investigated cells was from 12 to 29. Asterisks denotes statistical significance (Student's *t* test; P <0.05) between the Ca_V3 channel alone and Ca_V3 + β1b conditions. The voltage dependence of activation was estimated using a modified Boltzmann function to fit *I-V* data: $I = I_{max}(V_m - V_{rev})/(1 + exp((V_{50} - V_m)/k)$, where I means current, V_m is the test potential, V_{rev} stands for the apparent reversal potential, V_{50} is the mid-point of activation, and k means the slope factor. Steady-state inactivation (SSI) relationships were obtained by fitting averaged data to a standard Boltzmann function: $I = I_{max}/(1 + exp[(V_m - V_{1/2})/k])$, where I_{max} is the maximal current recorded at −30 mV, $V_{1/2}$ is the midpoint of steady-state inactivation, and k is the slope. When analyzing the time course of recovery from inactivation (τ_h) at −100 mV for Ca_V3 channels in absence and presence of β1b-subunit, the peak current values during the 50-ms pulse were normalized to the peak current at the 500-ms pulse. Then data were fitted by using a one-phase exponential association equation.
