# Supplementary material for: Increase of CaV3 channel activity induced by HVA β1b-subunit is not mediated by a physical interaction
Source: BMC Res Notes. 2018 Nov 14;11:810. doi: 10.1186/s13104-018-3917-1 (PMC6236959; doi:10.1186/s13104-018-3917-1)
Supplement: Supplementary file 2 — Additional file 2. Modulation by the β1b subunit is specific on CaV channels. Electrophysiological recordings and I-V relationship for HVA CaV1.2 and NaV1.6 channels in the absence and the presence of the β1b subunit. [file 13104_2018_3917_MOESM2_ESM.pdf]

Additional file 2.

Increase of Ca_V3 channel activity induced by HVA β1b-subunit is not mediated by a physical interaction

Rogelio Arteaga-Tlecuitl[1], Ana Laura Sanchez-Sandoval[1], Belen Ernestina Ramirez-Cordero[1], Margarita Jacaranda Rosendo-Pineda[2], Luis Vaca[2], Juan Carlos Gomora[1,*]

[1]Departamento de Neuropatología Molecular and [2]Departamento de Biología Celular y del Desarrollo, Instituto de Fisiología Celular, Universidad Nacional Autónoma de México. Ciudad de México, 04510, México.

[*]Corresponding author: jgomora@ifc.unam.mx

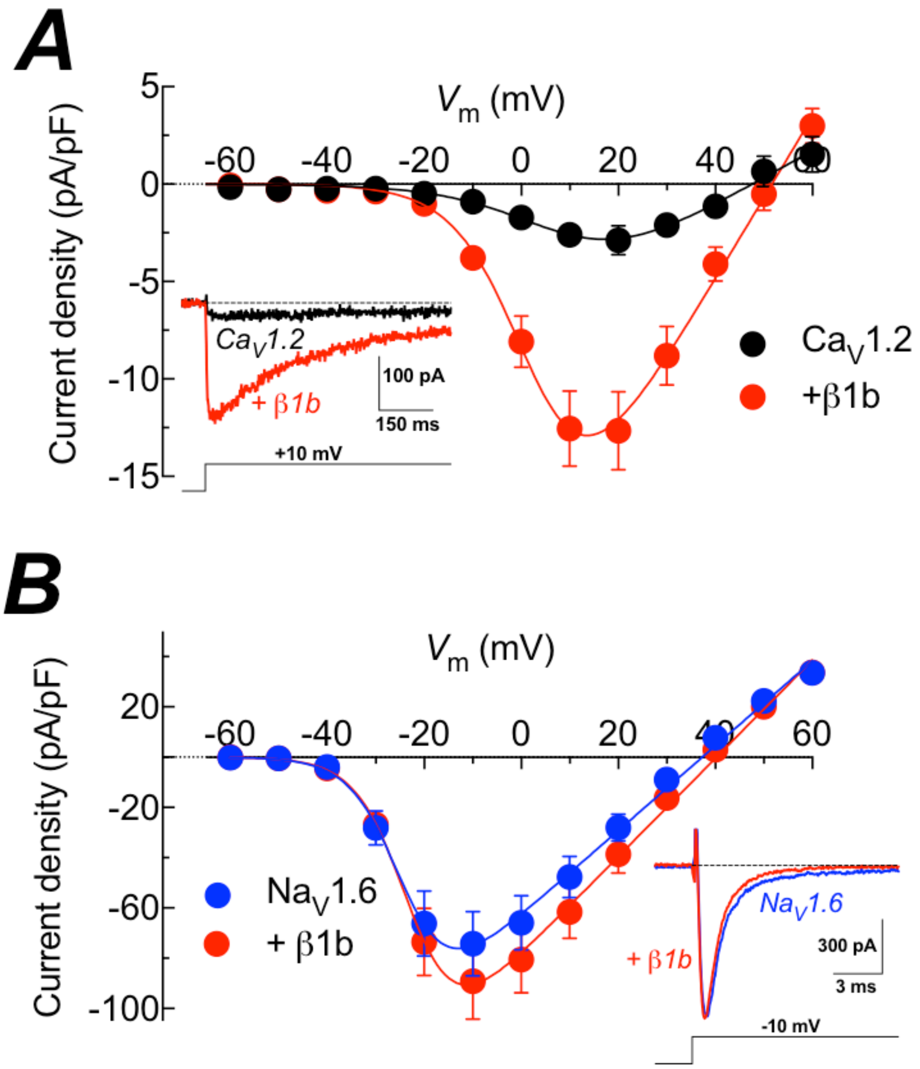

Additional file 2. **Modulation by the β1b subunit is specific on Ca_V channels**. ***A***, *I-V* relationship for HVA $Ca_V1.2$ channels in the absence and the presence of the β1b subunit, as indicated. Thirteen cells for $Ca_V1.2$ alone and 14 with the β1b subunit. *Inset:* representative whole-cell currents recorded at +10 mV from HEK-293 cells transfected with $Ca_V1.2$ channels alone or together with the β1b subunit. The charge carrier was 10 mM Ba^{2+}. ***B***, *I-V* relationship for $Na_V1.6$ channel in the absence and the presence of the β1b subunit. The number of cells investigated was 16 for both groups. *Inset:* representative whole-cell sodium currents recorded at -10 mV from HEK-293 cells stably expressing $Na_V1.6$ channels alone or together with the β1b subunit. The charge carrier was 158 mM Na^+. Patch-clamp experiments were performed using a HP of -100 mV. In both curves, data was fitted with a modified Boltzmann function (smooth lines). Notice that the regulatory effect of β1b subunit is specific for Ca_V channels, as $Na_V1.6$ channel current density was not significantly affected.
